# Vision and Visuomotor Performance Following Acute Ischemic Stroke

**DOI:** 10.3389/fneur.2022.757431

**Published:** 2022-02-16

**Authors:** Chamini Wijesundera, Sheila G. Crewther, Tissa Wijeratne, Algis J. Vingrys

**Affiliations:** ^1^School of Psychology and Public Health, La Trobe University, Melbourne, VIC, Australia; ^2^Department of Neurology, Sunshine Hospital, The University of Melbourne, Parkville, VIC, Australia; ^3^Department of Optometry and Vision Sciences, The University of Melbourne, Parkville, VIC, Australia

**Keywords:** acute ischemic stroke, eye-hand coordination, visual field, visual acuity-in-noise, vision, visuomotor function, Melbourne Rapid Field-Neural (MRFn), Lee-Ryan Eye-Hand Coordination Test (SLURP)

## Abstract

**Background:**

As measurable sensory and motor deficits are key to the diagnosis of stroke, we investigated the value of objective tablet based vision and visuomotor capacity assessment in acute mild-moderate ischemic stroke (AIS) patients.

**Methods:**

Sixty AIS patients (65 ± 14 years, 33 males) without pre-existing visual/neurological disorders and acuity better than 6/12 were tested at their bedside during the first week post-stroke and were compared to 40 controls (64 ± 11 years, 15 males). Visual field sensitivity, quantified as mean deviation (dB) and visual acuity (with and without luminance noise), were tested on MRFn (Melbourne Rapid Field-Neural) iPad application. Visuomotor capacity was assessed with the Lee-Ryan Eye-Hand Coordination (EHC) iPad application using a capacitive stylus for iPad held in the preferred hand.Time to trace 3 shapes and displacement errors (deviations of >3.5 mm from the shape) were recorded. Diagnostic capacity was considered with Receiver Operating Characteristics. Vision test outcomes were correlated with National Institutes of Health Stroke Scale (NIHSS) score at the admission.

**Results:**

Of the 60 AIS patients, 58 grasped the iPad stylus in their preferred right hand even though 31 had left hemisphere lesions. Forty-one patients (68%) with better than 6/12 visual acuity (19 right, 19 left hemisphere and 3 multi-territorial lesions) returned significantly abnormal visual fields. The stroke group took significantly longer (AIS: 93.4 ± 60.1 s; Controls: 33.1 ± 11.5 s, *p* < 0.01) to complete EHC tracing and made larger displacements (AIS: 16,388 ± 36,367 mm; Controls: 2,620 ± 1,359 mm, *p* < 0.01) although both control and stroke groups made similar numbers of errors. EHC time was not significantly different between participants with R (*n* = 26, 84.3 ± 55.3 s) and L (*n* = 31, 101.3 ± 64.7 s) hemisphere lesions. NIHSS scores and EHC measures showed low correlations (Spearman R: −0.15, L: 0.17). ROC analysis of EHC and vision tests found high diagnostic specificity and sensitivity for a fail at EHC time, or visual field, or Acuity-in-noise (sensivity: 93%, specificity: 83%) that shows little relationship to NIHSS scores.

**Conclusions:**

EHC time and vision test outcomes provide an easy and rapid bedside measure that complements existing clinical assessments in AIS. The low correlation between visual function, NIHSS scores and lesion site offers an expanded clinical view of changes following stroke.

## Introduction

Stroke is defined as sudden onset focal (or global) disturbance of cerebral function, lasting more than 24 hours, or leading to death, and with no apparent cause other than that of vascular origin (1). Stroke is recognized as one of the leading causes of adult mortality and disability worldwide, affecting over 16 million people annually with nearly five million deaths and another six million people developing permanent disability (2). Post-stroke recovery is often associated with persistent symptoms of impaired cognition, sensory and motor disability that tend to be accompanied by anxiety/depression and fatigue (3–7). Upper limb function is often impaired acutely (8–10) leading to reduced manual and coordination of visually guided motor tasks contralateral to the site of the brain lesion (3, 11). Clinical trials have reported that ~70% of referred stroke survivors post-hospitalization have ipsilaterally derived eye movement disorders (12–15) and reduced amplitude in micro-saccades which may affect visual sensitivity (16). We have also recently reported that ~2/3 of mild-moderate severity first episode acute ischemic stroke (AIS) patients with no previous history of impaired vision, experience deteriorated visual acuity-in-noise (VAn) with contralateral visual field defects immediately (i.e., within 7 days) after stroke (17). Given the ubiquity of motor involvement in stroke, we set out to examine and quantify visuomotor performance in the same group of hospitalized AIS cases whose sensory visual capacity was reported in the past (17), and for this study, we added a motor eye-hand coordination task. We also wanted to establish whether the visuomotor changes would associate with changes in conscious perception, sensory vision loss or the hemisphere of lesion (motor) and be reflected in the NIHSS scores. According to Lyden 2017 (18), NIHSS is the “most widely used deficit rating scale in modern neurology” and a well-validated and reliable relative measure of consciousness, limb motor capacity and oculomotor function (19). Finally, we aim to ascertain the value of these rapid tests as diagnostic agents of stroke.

Vision was quantified in terms of high contrast visual acuity (VA) in the absence or presence of luminous noise (visual acuity-in-noise) to ascertain impairments to central attention mechanisms (20). This was achieved with readily available Melbourne Rapid Field-Neural (MRFn) iPad application that can be used for bedside testing and quantification of vision deficits (described in an earlier publication) (17). Visuomotor function in terms of eye-hand coordination (EHC) has been established using the Lee-Ryan eye-hand coordination (SLURP) app, designed by the University of New South Wales (21). The SLURP app was designed to quantify trace time, accuracy and the extent of displacement when tracing three familiar shapes on an iPad. We hypothesized that in patients with intact central vision, hemisphere of lesion would limit hand of preference and that the time and number of errors during item tracing would be much greater in AIS patients compared to age similar controls. Our secondary aim was to consider the correlation of vision and visuomotor function as found with MRFn and the EHC task and NIHSS scores (22) which encompasses clinical rankings of sensory and motor function in AIS. Finally, we have calculated the diagnostic value of these vision tests in radiologically identified AIS.

## Materials and Methods

Ethics approval was provided by Sunshine Hospital (Western Health Ethics Committee HREC/16/WH/1) review board and the study was conducted in accordance with the tenets of the Declaration of Helsinki with all participants (or their carers) giving informed consent to participate. Clinical Trial registration: ACTRN12618001111268.

### Participants

**Figure 1:**
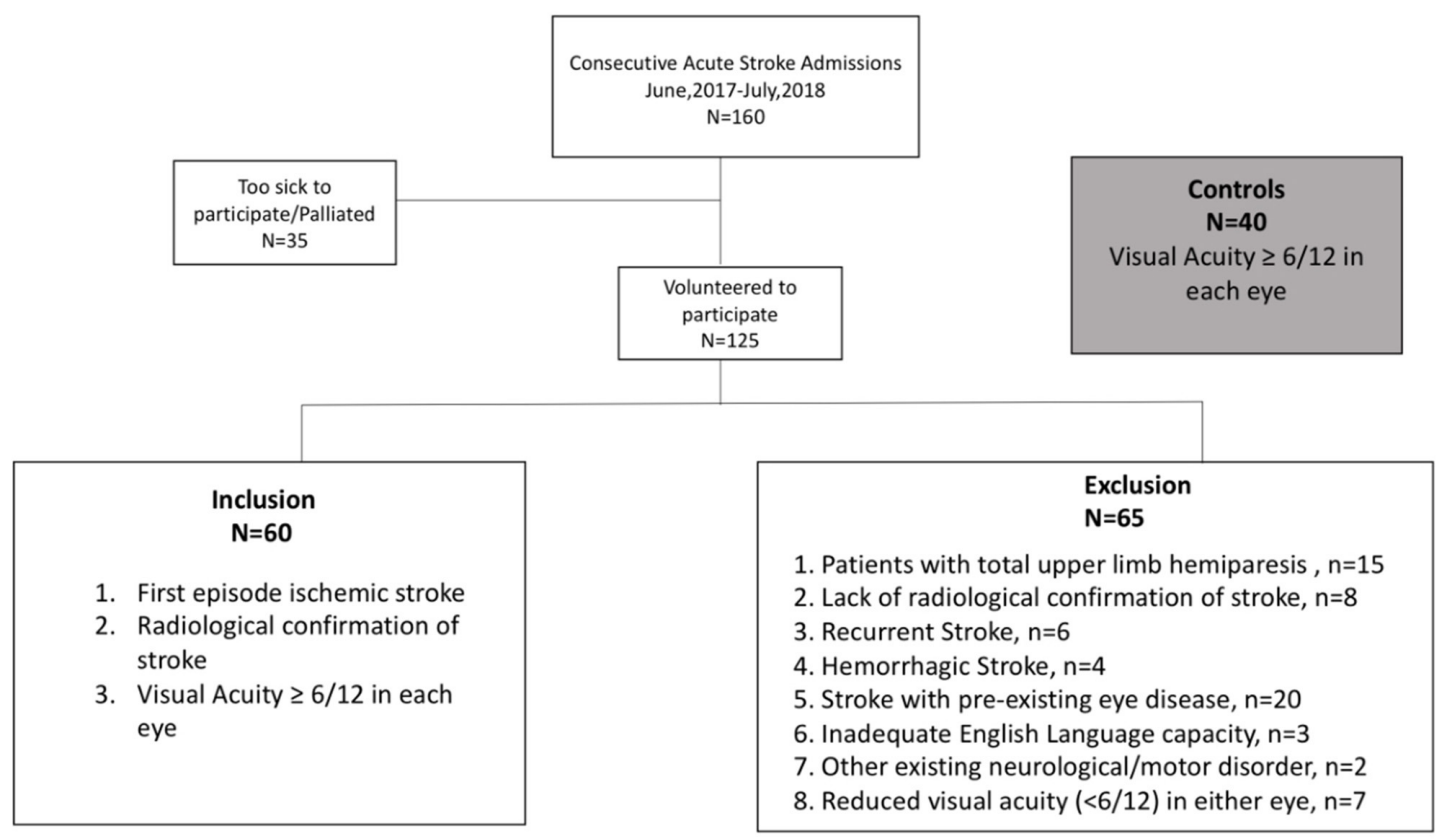
Consort flowchart including exclusion and inclusion criteria for the recruitment of the participants to the study.

One hundred and sixty (29–95 years, 68 ± 14.5years, 88 males) sequentially presenting, acute ischemic stroke patients admitted to Sunshine Hospital, Melbourne, between June 2017 and July 2018 were invited to volunteer for a subjective assessment of their vision and a quantifiable objective measure of visuomotor function. Those who agreed, and who met our inclusion criteria (see Figure 1) (17): first episode AIS, intact (better than 6/12) central high contrast visual acuity and radiographic confirmation of lesion site with no existing ocular/neurological disease or previous stroke, had their data analyzed for vision and EHC outcomes in this study. Sixty first-episode AIS patients (29–88 years, mean: 65 ± 14 years, 33 males) met our inclusion criteria and had their data analyzed for vision and EHC outcomes in this study whereas 65 others were excluded (Figure 1).

All testing was performed during the first week (usually day 2 or 3) of any participant's hospital stay. Fifteen stroke patients with total upper limb paresis were excluded as they were unable to lift their preferred arm or grasp the iPad stylus. Four out of the 20 excluded stroke patients had existing eye disease due to advanced macular degeneration with large central scotomas and VA <6/12. These participants could not see adequately to perform the EHC task. Two patients were excluded due to co-morbid, neuro-motor disorder and Parkinson's disease (PD). Each showed significant hand tremor consistent with PD while holding the pen. All other AIS patients were capable of a stable hand grip of the iPad stylus with their preferred hand (right hand 58/60) as needed for the tracing task (Figure 1).

Stroke diagnosis and severity was determined at the time of admission by a stroke neurologist. Site of the infarction and vascular territory was established and confirmed with routine computer tomography (CT/CT angiography) or magnetic resonance imaging (MRI/MR angiography) of the brain (23–25). This information was used to confirm the site and extent of the lesion and facilitate a structure-function analysis with the EHC measures (26). NIHSS scores of the patients in this study were recorded at the time of admission and confirmed by two stroke neurologists.

Patients wore their habitual reading glasses for testing and were examined binocularly at their bedside using the MRFn and SLURP iPad applications (17, 21). The adequacy of current reading glasses was established by measuring near visual acuity as 6/12 or better using the MRFn app.

Forty age-similar healthy controls (29–85 years, mean:64 ± 11 years, 15 males) were recruited following a comprehensive routine eye examination at an optometry practice of one of the authors (CW) after providing informed written consent for participation. These participants showed no evidence of current or past ocular, motor or neurological disorders, wore their habitual reading glasses for testing and provided normal near visual acuity (6/12 or better) prior to visuomotor assessment.

### SLURP (Lee-Ryan Eye-Hand Coordination Test) iPad Application

**Figure 2:**
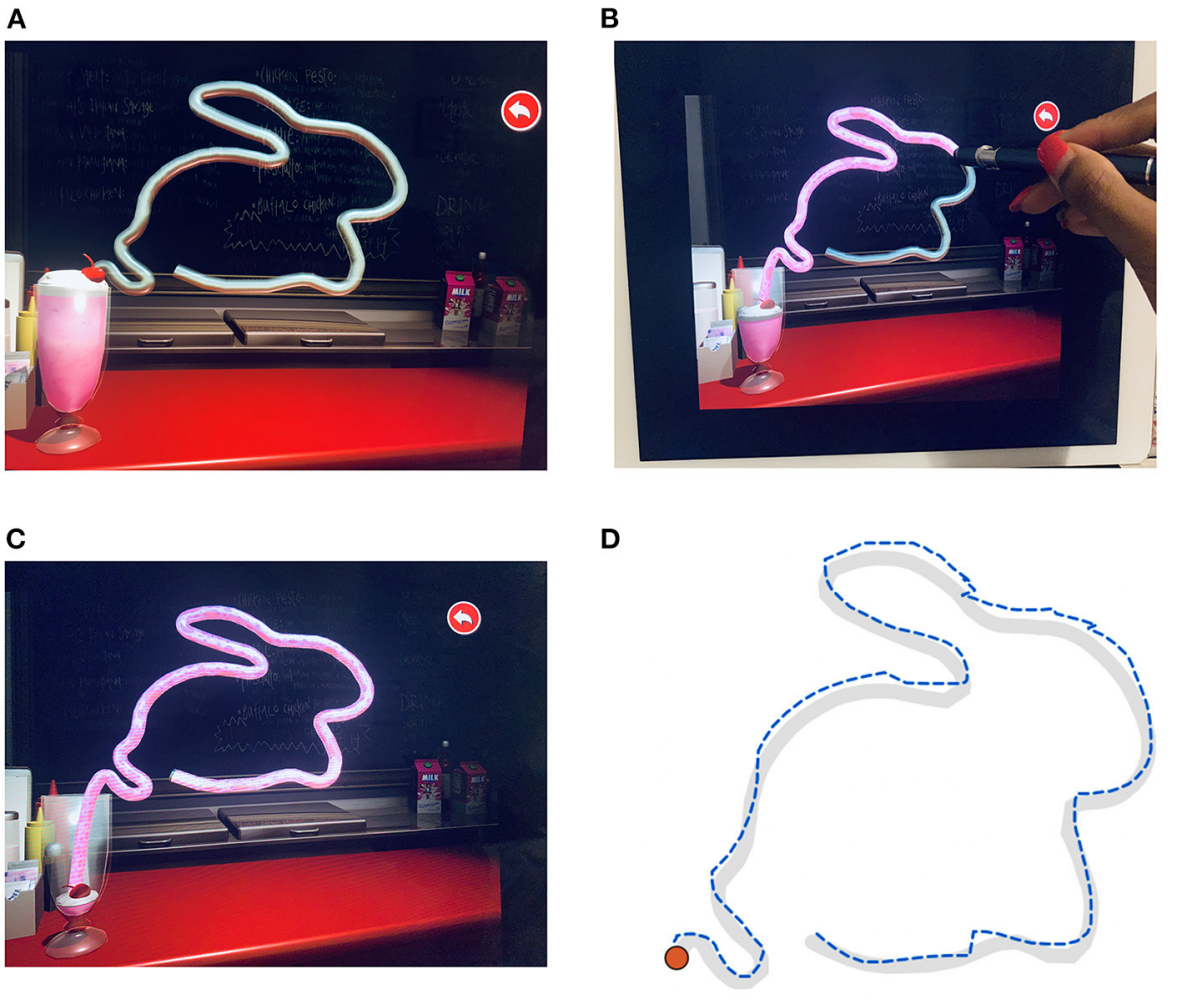
The UNSW Lee-Ryan Eye Hand Coordination Test (SLURP) using the rabbit shape. **(A)** Geometric shape of the rabbit prior to start of task. Timing is initiated when the participant touches the red cherry spot at the top of the glass carrying the milkshake of panel. **(B)** Example of Rabbit trace in progress with the stylus iPad pen. Note that the milk shake enters the straw and defines the path that has been correctly traced up to that time. **(C)** Completed trace of Rabbit shape. **(D)** Example of a patient's trace of the rabbit shape. The desired trace path is shown in gray. The blue dashed line identifies the participant's trace with displacement errors displayed as contour realignments.

The Lee-Ryan Eye-Hand Coordination (SLURP) test is available from Apple App Store for $2.10 USD—dated 13/08/2020—and was originally designed to provide a measure of visuomotor performance in children with amblyopia (21) on an iPad and in neurotypical adults whose results are similar to any of our age similar controls. We have chosen to use SLURP as a measure of EHC post-stroke due to its ease of use at a patient's bedside and evidence for its fast and sensitive assessment on a geriatric population (21, 27). As SLURP EHC test, is performed on a 2D setting, it is shown to assess attention and fine motor control with less demand on stereopsis compared to visuomotor tasks that involve grasping and bead threading where visually guided movements are adversely affected by eye movement impairments and visual deficits that reduce depth perception (28, 29). Hence SLURP EHC app objectively assesses both spatial and temporal accuracy of oculo-motor function that reflects cortical processing of visual attention and fine motor actions, in a generalized acute stroke population. There is no gold standard for measurement of EHC, however, previous studies (30–32) have suggested that the standard EHC measurement should involve the motor reaction time in any correlation of a visual task involving an interactive eye and hand modulating function in decision making. The methodology of EHC with SLURP subscribes to that. Other seminal EHC studies (33, 34) also use reaction time as the predominate marker of EHC assessment. Quantification of performance in SLURP is calculated by the time taken to trace a number of geometric or animal shapes shown on an iPad screen (Figure 2) and it is further measured by the number and nature of errors made while tracing each item (21). Tracing can be performed using an iPad stylus or by hand, with Junghans and Khuu (27) finding that the number of errors correlate with the type of stylus pen used for tracing. As a consequence, we adopted the Apple iPad rubber tip stylus pen (Capacitive Screen Touch Pencil Drawing Pen, for Apple iPad) in all cases to ensure consistency.

### Testing Procedures

Measurements of near visual acuity, visual acuity in the presence of luminance noise jitter and visual field sensitivity of all participants were performed monocularly in ambient binocular hospital room lighting using the Melbourne Rapid Field-Neural (MRFn) App where the methodology is detailed in a previous publication (17) and the outcomes are reproduced here with the approval of the editor of our past publication. For inclusion in the study, we required a near acuity better than 6/12 with habitual glasses (Figure 1) given that patients with worse near-acuity self-reported difficulty doing the EHC test. As the iPad is a calibrated source of light, external lighting has little impact on the visibility of targets (35) provided reflections off the screen are avoided. Screen brightness was set to maximum for 10 min prior to testing, to stabilize luminous output (36). All patients were asked to use their preferred hand to hold the Apple iPad pen (27).

The participants' task was to binocularly trace three shapes (circle, triangle and a rabbit) as quickly and as accurately as possible wearing normal near vision corrections and using the iPad stylus pen at a working distance of 33 cm with the iPad flat on a table (Figure 2A). Participants were told to initiate a trial by touching the “cherry shape” located at the top of the milkshake icon (Figure 2A). Verbal instructions regarding the task were given at the bedside and patients were requested to undertake a practice trial using the circle shape before commencement of formal testing to ensure they understood the nature of the task and were able to confidently and comfortably grasp the iPad stylus pen.

When the tracing is executed correctly (displacements within ± 3.5 mm) the straw outlining the shape becomes filled with “strawberry milk” (Figure 2B), thereby emptying the glass. Whenever the patient's tracing deviates from the shape path by >3.5 mm, the straw filling stops and a warning sound is activated. The patient was instructed to restart from where they last left off (end of milkshake in straw) as soon as possible after this sound and continue until they reach the end of the shape. The task algorithm returns time for completion, the number of deviation errors and the summed deviations beyond the 3.5 mm criterion.

### Data Analysis

Comparisons between stroke and control groups were made for visual acuity, visual acuity-in-noise, the visual field mean deviation (MD) and the average foveal threshold of the affected central visual field. Visual acuity and visual acuity-in-noise were measured using the MRFn application as detailed in a previous publication (17). The mean deviation is returned by a pointwise comparison of thresholds (dB) to age-normals, stored in the MRFn database. Foveal thresholds (dB) represent the average retinal sensitivity of four foveal points located at about 0.8 degrees from fixation in each quadrant. Although both eyes were tested for vision, the foveal thresholds associated with the affected hemifield in both eyes of AIS patients (hemifield contralateral to the CT/MRI defined lesion) were analyzed for the AIS group and compared to the findings returned from the right hemifield of all controls (comparison to the fellow hemifield does not change our findings).

The total time for completion of the three shapes, total number of errors and the total displacement during the tracing of the SLURP task were recorded and have been analyzed in conjunction with hemisphere of lesion and NIHSS score. The results have been compared to an age-similar control group (29–85 years, 64 ± 11 years, 15 males) as age has been previously highlighted as a significant factor affecting task performance (27). See Table 1 for descriptive statistics of controls.

**Table 1:** Summary of visuomotor and NIHSS score results for controls and AIS groups.

	**Controls** **(*n* = 40)**	**Stroke** **(*n* = 60)**	**R side stroke** **(*n* = 26)**	**L side stroke** **(*n* = 31)**	**Multi-territorial strokes (*n* = 3)**
Age mean (years ± months)	64 ± 11	65 ± 14	60 ± 14	69 ± 13	64 ± 11
Gender (M:F)	15:25	33:27	13:13	19:12	0:3
NIHSS Score	N/A	4.0 ± 4.0	4.2 ± 4.1	4.0 ± 4.2	2.7 ± 1.5
EHC time (s)	33 ± 11.5	93 ± 60.1 Fail: *n* = 40, *p* < 0.01	84 ± 55.3 Fail: *n* = 16, *p* < 0.01	101 ± 64.7 Fail: *n* = 22, *p* < 0.01	90 ± 59.4 Fail: *n* = 2
EHC errors	22.4 ± 8.8	24.6 ± 17.3 Fail: *n* = 13, *p* = 0.73	20.7 ± 11.8 Fail: *n* = 3, *p* = 0.63	26.6 ± 19.3 Fail: *n* = 8, *p* = 0.72	37 ± 32.0 Fail: *n* = 2
EHC displacement (mm)	2,630 ± 1,354	16,634 ± 36,625 Fail: *n* = 33, *p* < 0.01	13,682 ± 22,907 Fail: *n* = 15, *p* < 0.01	18,434 ± 46,324 Fail: *n* = 16, *p* < 0.01	23,621 ± 24,206 Fail: *n* = 2

*Fail identifies the number of AIS group beyond the 97.5th confidence limit of normal*.

Data for all variables are displayed as means and standard deviations with the 97.5th confidence limit of our age similar controls used as the criterion to identify “abnormal” outcomes for stroke participants. Statistical analysis and graphs were conducted using GraphPad Prism v7.00 for Windows www.graphpad.com. Cohort comparisons of visual capacity assessment in the form of visual acuity, field of vision and visuomotor spatio-temporal accuracy were made using a Student's *T*-test or non-parametric Mann-Whitney U test in cases where the data were not normally distributed. A conservative alpha value of 0.01 was employed to allow Bonferroni considerations. Correlations between variables were determined with non-parametric Spearman's rank correlation coefficient for our stroke population given the non-Gaussian distributions in all our data (visual acuity-in-noise, visual field mean deviation and EHC for controls and stroke cohorts) as The Kolmogorov-Smirnov test failed to find normality in controls and stroke groups. Igor Pro was used to produce multidimensional scatter plots. Receiver-operator-characteristics (ROC) were calculated to assess the diagnostic capability of each parameter.

## Results

Sixty of 160 (37.5%) mild-moderate (NIHSS Score: 4.0 ± 4.0) AIS participants were recruited and were able to perform the MRFn and EHC tasks at their bedside in a mean time of 7 and 2–5 min, respectively. Forty-one (68%) of the hospitalized AIS volunteers showed extensive visual field deficits. Of this group, 26 (43%) had right hemisphere lesions, 31 (52%) left hemisphere lesions and three participants (5%) had multi-territorial lesions. On average our patients had mild-moderate stroke manifestations as evident from the NIHSS scores (4.0 ± 4.0) for both Right (4.2 ± 4.1) and Left (4.0 ± 4.2) sided lesions (Table 1).

Of the 26 with right hemisphere lesions, 19 (73%) had visual field deficits, which included 16 left hemianopias (84% of the visual field deficits), 1 left quadrantanopia and 2 diffuse altitudinal losses. Among the 31 left hemisphere lesions, 19 (61%) showed visual field deficits with 13 having right hemianopias (68% of the visual field deficits), 2 right quadrantinopias and 4 diffuse altitudinal losses.

### Visuomotor Performance in Terms of Time and Errors Made on EHC-SLURP Task

Despite significant hand motor limitations as assessed on NIHSS in 10/60 patients with a NIHSS score >9, only 2/10 were found not to choose to pick up the stylus with their previously dominant right hand. Both had restricted dominant arm movement post-stroke. One was radiologically defined with a frontal lobe infarct, in the areas around Broca's area in their left hemisphere and the other, had a lesion in the right hemisphere middle cerebral artery causing a posterior territory infarction.

**Figure 3:**
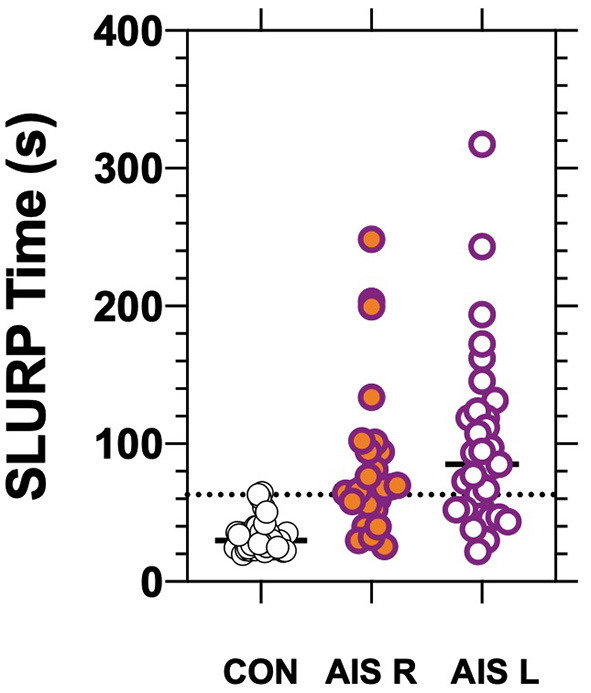
Eye-hand coordination time for controls and AIS patients having Right (*p* < 0.01) or Left (*p* < 0.01) hemisphere lesions as confirmed radiologically. The bars show group/individual tracing time and the horizontal dotted line indicates the 97.5th percentile for control.

The stroke patients completed tracing of the three shapes (circle, triangle, rabbit) with an average total time of 93 ± 6 0.1 s. Control patients required a significantly (*p* < 0.01) shorter average time of 33 ± 12.8 s to complete tracing of the same shapes in the same order (Figure 3). Both the circle [AUC: 0.92, (CI: 0.87–0.97), *p* < 0.01] and rabbit [AUC: 0.89, (CI: 0.82–0.95), *p* < 0.0001] show similar areas under the ROC curve. Although a significant increase in tracing time was found for each EHC task, we did not find a statistically significant difference in tracing time or tracing errors related to hemisphere of lesion (see Table 1; Figure 3).

Surprisingly, our EHC results find that the AIS and control groups make a similar number of average errors (controls: 22 ± 9; stroke: 25 ± 17, *p* = 0.95) during the tracing of all three shapes. Stroke patients, however, give significantly (*p* < 0.01) greater average displacements per error than controls (controls: 117.6 mm/error; stroke: 677.6 mm/error). As a consequence, stroke patients made significantly (*p* < 0.01) larger cumulative displacements (mm) during the tracing of the 3 shapes (16,388 ± 36,367 mm) relative to controls (2,620 ± 1,359 mm) consistent with their longer tracing times. Furthermore, stroke patients take significantly longer to return to the correct spatial location and restart tracing when they make an error (displacement/time: controls: 79.4 mm/s, stroke: 178.0 mm/s, *p* < 0.01). The total time taken for the completion of these tests by stroke patients showed a moderately positive correlation with both displacement (Spearmann's *r*: R: 0.78, L: 0.74) and number of errors (Spearmann's *r*: R: 0.66, L: 0.79) for cases with right or left hemisphere lesions.

### Relationship Between NIHSS Scores and Vision and Visuomotor Function

**Figure 4:**
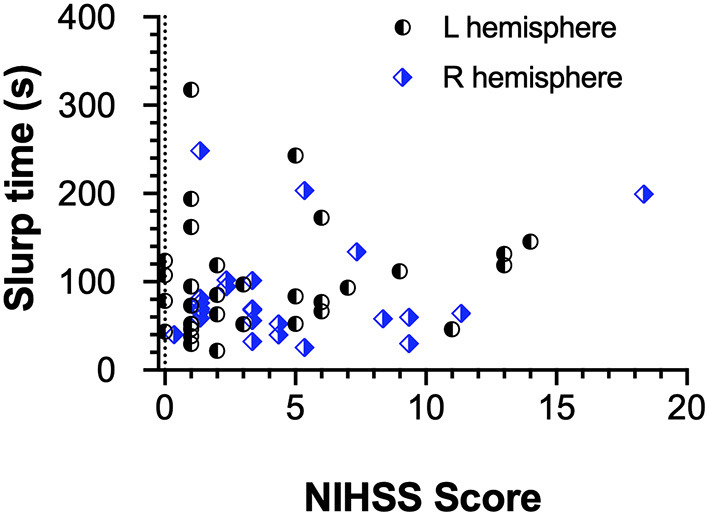
EHC Time as a function of NIHSS Score and Hemisphere of lesion. The scatter plot shows a poor correlation between both parameters regardless of the affected hemisphere.

As NIHSS scores reflect somato-sensory and motor capacity we compared these scores with vision outcomes and the total time taken for completion of the EHC tasks for all three shapes. The Figure 4 demonstrates that most stroke patients with low NIHSS scores, perform the EHC in a shorter duration. However, statistically our results (Figure 4) show a low correlation between EHC total time and NIHSS score (*r* = 0.09) for the entire group and for the cohorts in terms of left or right hemisphere lesions (Spearman's R −0.15, L 0.17). Similarly, poor correlations were found for NIHSS scores and EHC errors (R −0.03, L 0.19) and NIHSS scores and total displacement (R 0.15, L 0.31) with both right and left hemisphere lesions respectively.

**Figure 5:**
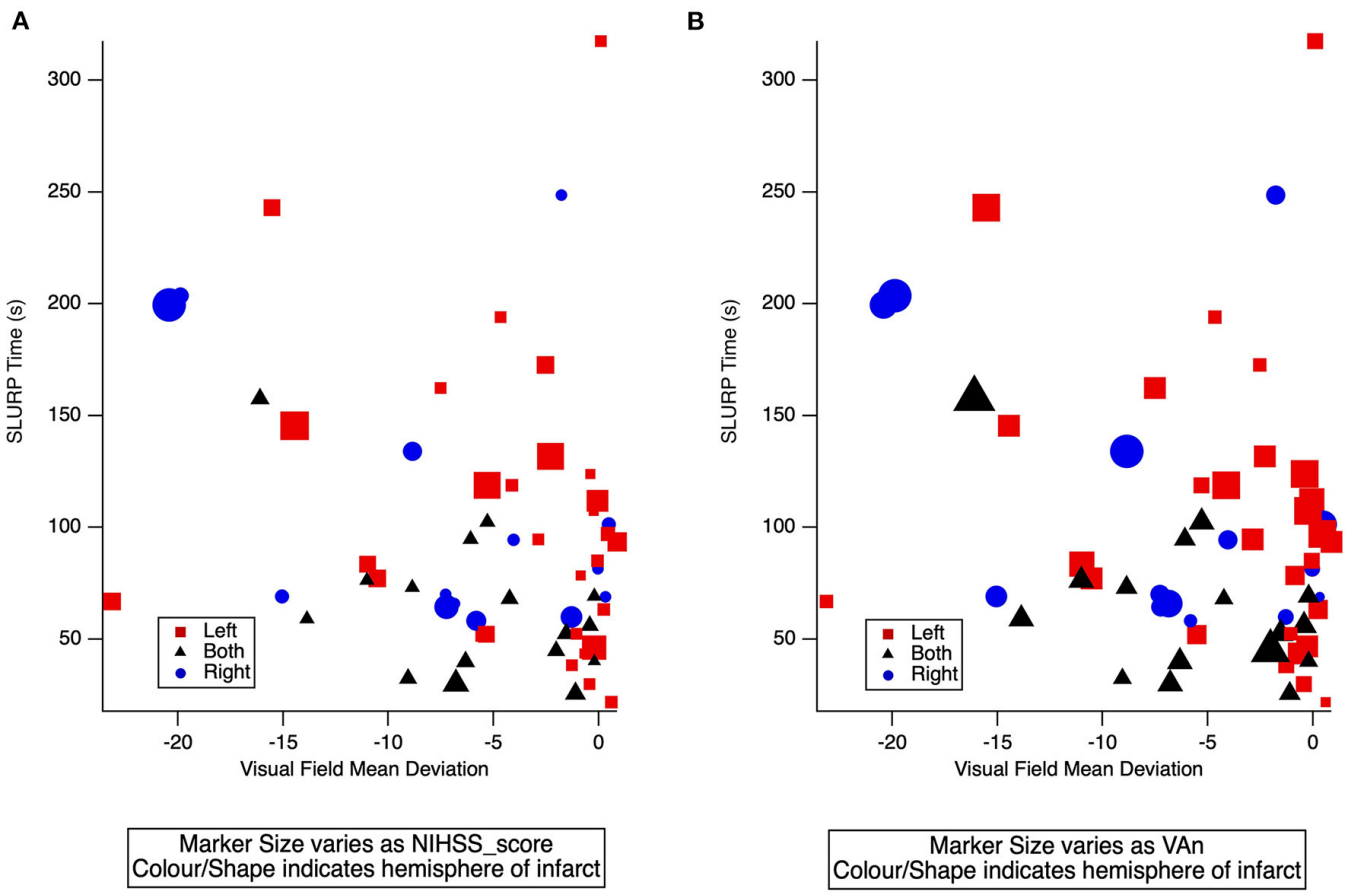
**(A)** 3-D scatter plot of individual AIS patient EHC (SLURP) time and mean deviation of visual field sensitivity covarying with NIHSS score. **(B)** 3-D scatter plot of individual AIS patient EHC (SLURP) time and mean deviation of visual field sensitivity covarying with visual acuity-in-noise (VAn). Blue Circle: Right Hemisphere lesion, Red square: Left Hemisphere lesion, Black Triangle: a lesion in both hemispheres. Lesion site and severity of AIS is indicated by shape, color and size of spot respectively.

The NIHSS scores with visual acuity-in-noise (*r* = 0.1) and visual field loss (*r* = −0.3) return low correlations. Figure 5 confirms the lack of correlation between mean deviation, EHC (SLURP) time and NIHSS score in Figure 5A or with Visual Acuity-in-Noise (VAn) in Figure 5B. It also demonstrates that the lesion location (hemisphere) does not mediate these processes.

### The Role of Central Foveal Threshold on Visuomotor Performance

Although both the AIS and control groups had average high contrast visual acuity of 6/7.5, 62% of the AIS group showed a significant deterioration to 6/12 with visual acuity-in-noise whereas controls remained unaffected by the presence of luminance noise (17). Hence, as visual acuity-in-noise is a foveal task, we considered whether foveal thresholds might influence hemianopia. Significantly reduced foveal thresholds regardless of the side of the lesion (R = 18.4 dB and L = 18.0 dB) were present. However, EHC time in 13/19 left hemianopias and 16/19 right hemianopias return strong negative correlations with their EHC time (Spearmann's R: −0.57, L: −0.42) indicating that a reduced hemi-field sensitivity may underlie the poorer EHC task.

### Lesion Effects on Expected Structure Function Relationships

**Figure 6:**
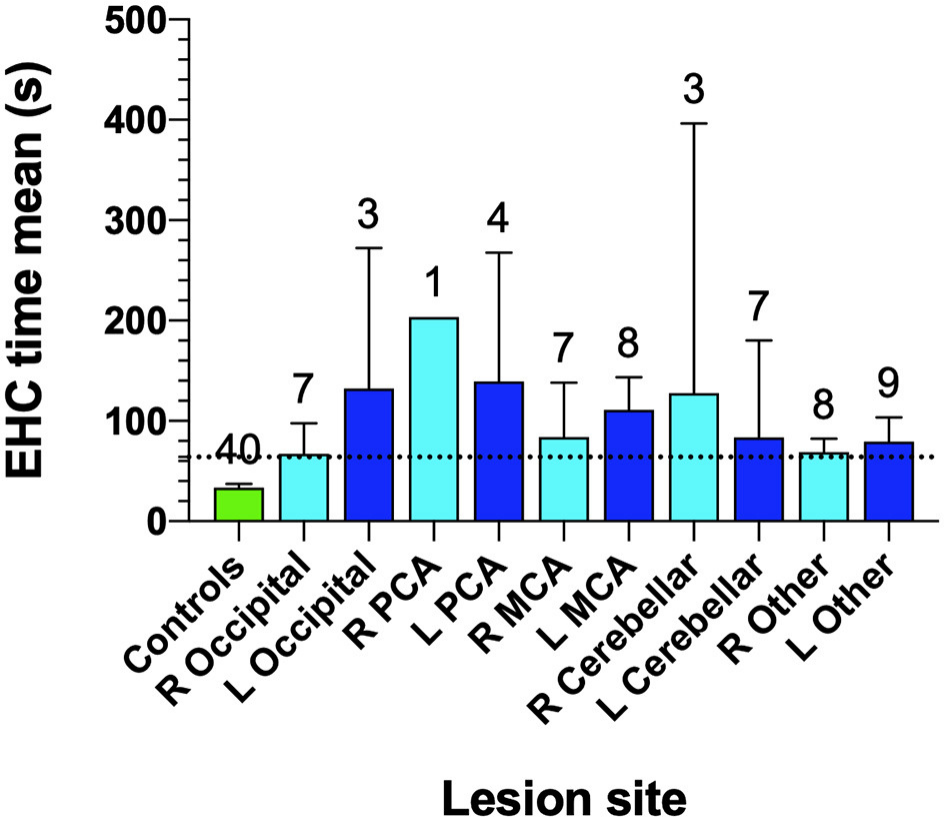
Eye-hand coordination for controls and site of lesion in AIS patients. Cohort number is shown at the top of each bar. Bars identify mean EHC time for various lesion locations and error bars are SD. The horizontal dotted line identifies the 97.5th percentile for controls.

The structure-function relationship between stroke and area of infarction was analyzed in terms of hemispheric localization of the stroke lesion and the time taken for completion of the EHC tasks (Figure 6). Surprisingly we find that lesions in most brain regions yield increases in tracking time for EHC performance (Figure 6). Our findings do not show any significant correlations between the hemisphere of lesion and the number of errors or the displacements made during the EHC task.

In Figure 6, the least impairment in EHC performance was found in our occipital lesion (*n* = 7, 67.1 ± 33.0 s) group in the right hemisphere, followed by “other lesion” cohorts in the right side (*n* = 8, 68.9 ± 16.3 s) which included 4 frontal lobe lesions, 3 parietal lobe lesions, 2 basal ganglia lesions, 2 pons lesions, 2 anterior cerebellar artery lesions, 1 internal carotid artery lesion, 1 prefrontal lesion, 1 internal capsule lesion and 1 corona radiata lesion.

### Value of Vision Tests in Identifying Cases of Early Stroke

**Figure 7:**
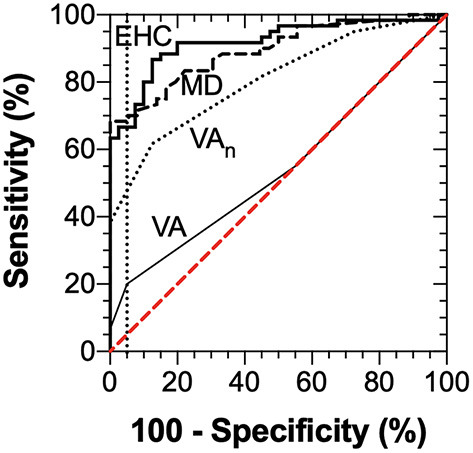
ROC Analysis for visual acuity (AUC: 0.54, 95% CI: 0.43–0.66), visual acuity-in-noise (AUC: 0.81, 95% CI: 0.72–0.89), Mean Deviation of the visual field (AUC: 0.89, 95% CI: 0.83–0.95) and Eye-hand coordination (AUC: 0.92, 95% CI: 0.86–0.97) time.

Diagnostic accuracy of the MRFn visual field mean deviation, EHC time from the SLURP app, visual acuity (VA), visual acuity-in-noise and EHC time were evaluated by considering Receiver Operator Characteristics (ROC) (Figure 7). The ROC analyses shows a diagnostic specificity (98%) and sensitivity (67%), (AUC: 0.92, 95% CI: 0.86–0.97) for EHC time. Visual field mean deviation (AUC: 0.89, 95% CI: 0.83–0.95, has sensitivity: 68%, specificity: 94%). Visual acuity-in-noise returns a moderate AUC (AUC: 0.81, 95% CI: 0.72–0.89), with sensitivity (62%) and specificity (88%) whereas high contrast visual acuity had no diagnostic capacity in our mild stroke cases (AUC: 0.54, 95% CI: 0.43–0.66, sensitivity:20%, specificity:95% with performance that encompassed chance (diagonal line). Applying three tests (EHC time, Mean deviation, Acuity-in-noise) with an “OR” logic gives sensitivity (93%) and specificity (83%).

## Discussion

### Vision Outcomes

As sensory-motor processing is a core diagnostic feature of AIS, vision and visuomotor capacity have been ascertained and quantified in 60 first event AIS patients with no prior history of visual anomalies during their first week of hospitalization. Assessments were made using the rapid SLURP EHC task (<2 min testing time) and the MRFn (<7 min testing time) apps. Although 41/60 of stroke sample showed significant visual field deficits, 37/60 presented a significant deterioration in visual acuity-in-noise and 40/60 showed a significant deterioration in the time taken to complete the visuomotor task and 18/41 with visual field deficits were unaware of any loss in vision. The presence of quantifiable visual deficits in 2/3 of AIS stroke population stand in line with the findings from previous studies where 92% of the 915 population of stroke patients referred for suspected vision problems, tested at a median of 22 days post-stroke presented some form of visual deficit, with the commonest being visual field deficits followed by visual attention deficits and eye movement deficits (13, 37).

### Visuomotor Performance and the Role of Central Foveal Threshold on Visuomotor Performance

With our AIS cohort, 58/60 patients were capable of picking up, grasping, holding and using the iPad stylus pen in their preferred hand. They chose to use their previously dominant right hand even though 31/60 had confirmed lesions in the contralateral left hemisphere. Overall the AIS group were all able to trace the three prescribed shapes but the mean time for completion of the three items was significantly longer for the stroke group than the age and vision matched healthy controls. Surprisingly the number of errors in tracing was similar for the stroke group to that of controls. However, the displacements per error of the stroke group were significantly larger and required longer time to relocate the stylus back to the task irrespective of hemisphere of lesion. See results illustrated in Figure 4.

Our results are consistent with previous studies that have shown that stroke patients require longer times to perform eye hand coordination tasks (3, 38, 39). Similar increases in time to perform a visuomotor task correctly has also been described in acquired traumatic brain injuries (40) and in the presence of impaired cognition in stroke (34). Such observations have been further associated with impaired proprioceptive judgments (41, 42) though previous results were predominantly considered for upper arm power and reaching action of the hand.

Our findings, for the same numbers of errors but significantly different displacements and relatively longer time to relocate to the pink “straw” path task of stroke patients compared to controls, is consistent with prior research showing that effective ocular-motor coordination requires optimal synergistic sensorimotor function of vision and proprioception (3, 43–45). Our findings also suggest that accuracy of motor control for familiar automatic routines is not grossly disturbed but rather that time and capacity to correct displacements following error requires conscious attention. Interestingly, our results would seem to indicate that optimal field of binocular vision was not entirely necessary to prevent tracing errors as 41/60 of our patients had quite severe visual field deficits with 31 having left hemisphere lesions. Previous research has demonstrated substantial foveal sparing (16) in the presence of hemianopia in stroke patients. However, consideration of foveal threshold sensitivity within the impaired visual field, i.e., hemianopia both on right and left, is significantly reduced with patients showing ~18/30 dB visual field sensitivity and impacts on the timing to complete the EHC tasks.

### Relationship Between NIHSS Scores and Vision and Visuomotor Function

Ten out of the 60 AIS patients demonstrated moderate motor impairment (46) with NIHSS scores >9. Of these 10 patients, 9/10 showed significant visual field deficits, 7/10 took significantly longer time for visuo-motor performance and 6/10 showed impaired visual-acuity-in noise. The low correlation between the NIHSS scores and EHC was unexpected and may be related to our small sample though the choice of all left hemisphere lesions patients to use their formerly preferred right hand irrespective of hemisphere of lesion argues against this. Rather the difference may be more related to the type of motor task and degree of familiarity and automatization associated with the manual movements requirements. Indeed our EHC tasks require well learnt automatized hand motor control rather than the NIHSS motor assessment requirement for voluntary control and organization of upper arm muscle power. Furthermore, NIHSS score encompasses ranking of sensory and motor function with prominent emphasis on motor components whereas EHC predominantly involves the fronto-parietal network with goal oriented fine motor action (3, 33) and less upper arm movement with the SLURP testing (21).

### Lesion Effects on Expected Structure Function Relationships

Our findings indicate that right handed patients with left hemisphere lesions did not take significantly longer to complete EHC tracings than did right hemisphere lesion patients were most unexpected. Indeed, the significantly longer time for both the left and right hemisphere stroke patients to trace and complete a shape after deviating outside the outline suggests that stroke patients require a longer time to consciously re-plan and reprogram the recovery path than does motor control *per se* specially for those with right hemisphere lesions given that the right frontoparietal network is known to play a greater role in attentional control of goal directed planning (47–49), and visuomotor actions. This interpretation was supported by Figure 5 that shows the lack of correlation of EHC time with the NIHSS scores or hemisphere of lesion in our AIS participants.

An associated interpretation for our findings could be that the increased time taken in tracing the shapes in the “SLURP” EHC tasks, may reflect a generalized impairment in visually driven attention and conscious reprogramming of spatial relocation processing needed for visuomotor actions, as a direct result of an impairment in the fast conducting magnocellular driven foveal vision rather than stroke damage to motor function (50). Such an explanation is supported by the observation that as expected (13) the 5 patients with posterior cerebral artery (PCA) lesions and possible primary visual cortex and dorsal visual stream damage (1 Right hemisphere,1 Left occipital and 4 left PCA lesions) took greatest average durations to complete the item tracings (Figure 4) while most other stroke patients, irrespective of the site of the lesion or the hemisphere, required a similar amount of time to complete the tracings, which was significantly longer than control but less than in the PCA cases. Such a generalized AIS induced impairment in EHC further raises the possibility that widespread hemispheric oedema (51) in the days following an AIS episode is a contributing source of impairment for sensory-motor integration and reduced attention required for fast and precise previously automatic EHC (52).

### Value of Vision Tests in Identifying Cases of Early Stroke

In terms of translatable findings and the diagnostic value of tablet based bedside vision tests in acute stroke management (18), our analysis of the ROC measures (Figures 3, 4) indicate that the “time” taken to perform the tracing of the three SLURP items gives the most sensitive measure of visuomotor compromise. Both time and the “extent of the displacements” provide useful quantifiable measures of stroke and visuomotor performance in relation to hand stability (21) of the EHC task irrespective of hemisphere of lesion. In addition, our findings demonstrate that the visual acuity-in-noise, visual field deviation and EHC are better discriminants in aiding the diagnosis of acute stroke than the more generally used high contrast visual acuity which shows little diagnostic capacity.

Individual performance in tracing the three shapes has also been compared to determine if one shape might be more useful diagnostically than the other two. Interestingly, duration for circle tracing shows moderate sensitivity (57%) at a specificity of 95% in detecting an abnormality in EHC in relatively short time of 35.0 s in stroke patients at high correlations with both R (Spearmann's *r* 0.83) and L hemispheres (Spearmann's *R* 0.80). Both the circle and rabbit) show similar areas under the ROC curve indicating high diagnostic capacity. Thus, it would appear that using a single shape (rabbit or circle) could provide excellent diagnostic capacity with minimal test time, making this a clinically useful and rapid measure of visuomotor impairment. Such a rapid assay might be useful in home measurements and potentially in the ambulance and emergency department by the non-stroke team clinicians with added value to complement and aid in the diagnostic process. Recent studies have shown increased EHC deficits in the presence of hemianopias from stroke (53). We do not however claim that our findings of EHC deficits shown in SLURP are exclusive to stroke as we have not made comparisons with other similar groups such as acquired brain injury, Parkinson's disease, migraines and TIAs although not all acquired brain injury conditions are as likely to affect vision in the same way as for stroke patients.

## Limitations

The generalizability of this study is limited by the size of the sample of mild-moderate AIS patients in a hospitalized environment that did not encourage testing of both hands to determine the effect on the non-dominant hand and contralateral hand. Most particularly small sample size made it difficult to compare hemispheric and lesion site effects on EHC duration in the hospital environment. A further limitation to our study is the lack of independent cohort groups such as migraine, other motor compromised disease such as Parkinson's disease, acquired brain injury and TIA patients to ascertain the ability of SLURP app to differentiate a stroke from such conditions that mimic stroke. Patient fatigue also limited testing of monocular as well as binocular effects of AIS on SLURP performance. A further limitation of the assessment of visuomotor function with the SLURP App is that the App operates in a two-dimensional plane which requires fine motor control rather than voluntary upper arm function and so is less of a test of upper arm movement and motor power than the holding of an arm extended palm down as undertaken with the NIHSS task. In addition, the SLURP App cannot be used in the presence of total upper limb paresis. On the other hand, SLURP app allows testing of focussed and sustained attention, ability to conform the hand to pick up and grasp the stylus and sophisticated manipulation of the hand and arm to enable tracing of the shapes. A further limitation to the theoretical interpretation is the lack of functional imaging, such as fMRI, to assess the extent of activation in visual attention networks during the performance of the impaired visual fields and during SLURP tracing by patients. However, despite these limitations, the translatable importance of the two Apps should be recognized in that the SLURP task is a sensitive quantifiable measure of the hand/grasp construct as in traditional EHC tests. The MRFn allows assessment of the most important sensory information in the form of vision that drives most cognitive and motor behavior and occupies the largest cortical and subcortical brain volume (48, 54).

### Future Work

Further longitudinal follow up will be required to define the mechanisms and underlying structure-network-function relationships in visuomotor function in AIS. Large scale, multi center studies will be required to further validate and longitudinally monitor recovery of visuomotor function following stroke to establish relationships between more regionalized lesions, and impairment in the goal directed parieto-frontal network activated during EHC tasks. Future studies with stroke mimics such as Parkinson's, migraines and TIA patients would also be required to explore the diagnostic value of SLURP in acute mild-moderate stroke.

## Conclusion

Our findings and the methodology of the testing, demonstrate the translatable value of the iPad apps that can rapidly and sensitively quantify relative timing of residual visual capacity and motor sensory integration. In particular the SLURP app measures EHC that requires eye movement planning, visuomotor planning and executive functionality to achieve goal directed actions post-stroke. The MRFn and SLURP apps are patient friendly and together provide easy and fast (<9 min in total for most patients) clinical tools, that can be used to quantify and assess multiple brain functions required to plan, control and execute fine motor tasks following acquired brain injury such as stroke and could be used to monitor their recovery.

Our findings also demonstrate that EHC deficits occur irrespective of lesion site across most areas of the brain without a structure specific component indicating that AIS patients with good visual acuity (>6/12) do not show the expected specific lesion structure-function impairment with regard to residual visuomotor capacity. Indeed our results suggest that the ubiquitous cerebral dysfunction/oedema, that accompanies AIS (52), is likely to contribute significantly to many of the acute acquired deficits including ability to attend, planning movements and cognitive and affective status (6, 55, 56).

## Data Availability Statement

The raw data supporting the conclusions of this article will be made available by the authors, without undue reservation.

## Ethics Statement

The studies involving human participants were reviewed and approved by Sunshine Hospital, Western Health Ethics Committee, Melbourne, Australia, HREC/16/WH/1. The patients/participants provided their written informed consent to participate in this study.

## Author Contributions

CW was involved in planning, design of the experiments, was responsible for recruitment of patients and all aspects of data collection, contributed to analysis of the data, prepared figures and tables, authored and reviewed the paper, and approved the final draft as part of her doctoral research. TW as head of Hospital Department of Neurology managed ethical concerns, facilitated patient access and recruitment, led acquisition and interpretation of all radiological data, NIHSS scores, and contributed to drafting of manuscript and final approval. AV contributed to design of experiments, led data analysis, preparation of figures and interpretation of visual field results, co-authored and reviewed drafts of the manuscript, and approved the final version. SC conceptualized, designed and funded the study *via* internal grants, contributed to analysis, theoretical interpretation of the data, and drafting of manuscript and final approval. CW, AV, and SC had full access to all the data in the study. All authors contributed to the article and approved the submitted version.

## Conflict of Interest

AV is a founding director of Glance Optical Pty Ltd., the maker of Melbourne Rapid Field-Neural (MRFn) App. The remaining authors declare that the research was conducted in the absence of any commercial or financial relationships that could be construed as a potential conflict of interest.

## Publisher's Note

All claims expressed in this article are solely those of the authors and do not necessarily represent those of their affiliated organizations, or those of the publisher, the editors and the reviewers. Any product that may be evaluated in this article, or claim that may be made by its manufacturer, is not guaranteed or endorsed by the publisher.

## Supplementary Material

Supplementary Table S1 Raw data for controls and stroke participants.

## Abbreviations

MRFn App: Melbourne Rapid Field Neural App
SLURP: Test Lee-Ryan Eye-Hand Coordination Test
EHC: Eye-Hand Coordination
VA: Visual Acuity
Van: Visual Acuity-in-Noise.
